# The Diagnostic Value of Point-of-Care Ultrasonography in the Differential Diagnosis of Azoospermia: Introducing a Concept

**DOI:** 10.3390/jcm14082837

**Published:** 2025-04-20

**Authors:** Shlomi Barak, Netanel Waldenberg, Guy Bar, Oshri Barel, Snir Dekalo

**Affiliations:** 1 Reproductive Services-Obstetrics and Gynecology, Assuta Ashdod University Hospital, Ashdod 6134789, Israel; 2 Obstetrics and Gynecology, Faculty of Health Sciences, Ben-Gurion University of the Negev, Beer Sheva 8410501, Israel; 3 The Multidiciplinary Center for Female and Male Infertility, Tel Aviv 6422521, Israel; 4 Obstetrics and Gynecology, Assuta Ashdod University Hospital, Ashdod, Israel, Affiliated with the Faculty of Health Sciences, Ben-Gurion University of the Negev, Beer Sheva 8410501, Israel; 5 Urology Department, Tel Aviv Sourasky Medical Center, Faculty of Medical Health and Sciences, Tel Aviv 6423906, Israel

**Keywords:** POCUS, NOA, OA, epididymis

## Abstract

**Purpose**: The aim of this study was to investigate the effectiveness and reliability of point-of-care ultrasonography (POCUS) in the differential diagnosis of azoospermia. **Materials and methods**: Records of 175 patients who had previously been diagnosed with normal-volume, normal-PH azoospermia and who had undergone surgical sperm retrieval were reviewed retrospectively. Patients’ preoperative evaluations included a comprehensive history and physical examination and a routine scrotal POCUS performed during their initial consultation by a non-radiologist treating andrologist in a clinic setting. Positive scrotal imaging revealed ectasia of the rete testis and/or dilation of the epididymal ductules. Based on their preoperative assessments, patients were guided to undergo either testicular sperm aspiration (TESA)/microsurgical sperm aspiration (MESA) procedures for those with suspected obstructive azoospermia (OA) or microdissection testicular sperm extraction (micro-TESE) for those with suspected non-obstructive azoospermia (NOA). **Results**: Of the 175 patients, 58 patients had normal follicle-stimulating hormone (FSH) levels (≤12 IU/L) and normal testicular volume. Thirty of them had no secondary signs of obstruction in their scrotal POCUS and subsequently underwent micro-TESE. All were confirmed to have NOA. Twenty-eight patients demonstrated at least two secondary signs of obstruction on scrotal POCUS. Of these, 15 underwent TESA, and 13 underwent MESA procedures. Twenty-seven patients were confirmed to have OA, and one was confirmed as having NOA. Among this cohort of men, the sensitivity of scrotal POCUS in diagnosing OA was 100%, whereas the specificity was 96.8%. Positive and negative predictive values (PPVs and NPVs) were 96.4 and 100%, respectively. **Conclusions**: Scrotal POCUS is an effective clinical diagnostic tool for distinguishing obstructive and non-obstructive azoospermia. Being noninvasive, safe, and affordable makes it an ideal bedside clinical tool that can serve the skilled non-radiologist clinician reliably.

## 1. Introduction

Azoospermia is defined as the total absence of spermatozoa in the ejaculate. It is present in 10% to 15% of men who undergo fertility investigation [1].

The classification of azoospermia into obstructive (OA) and non-obstructive (NOA) has major clinical relevance because it dictates patient management and affects the type of treatment required [2].

Hence, the differential diagnosis between OA and NOA is the first step in the clinical management of such patients, and it is usually based on a detailed medical history, physical examination, semen analysis, hormonal assessment, genetic testing, and imaging studies [3].

Due to the importance of differentiating between OA and NOA, AUA/ASRM guidelines even consider a testis biopsy in men with normal follicle-stimulating hormone (FSH) levels and testicular volume to determine the etiology [4].

The diagnosis of azoospermia can be a devastating experience for the infertile patient or couple, who frequently express their emotional distress while attending their initial and often their second-opinion fertility consultation [5].

Distinguishing between obstructive and non-obstructive azoospermia **early** in the diagnostic process, preferably at the time of initial consultation, may assist the clinician in alleviating obstructive azoospermia patients’ distress and in directing them more accurately towards their most probable diagnosis.

Point-of-care ultrasonography (POCUS) is becoming a very important, accurate bed-side tool in many situations and different disciplines [6]. It is easy to use and adds clinical information to the exam. However, in the setting of azoospermia, its added value in detecting obstruction has never been documented.

Therefore, the aim of this study was to investigate the effectiveness and reliability of POCUS at the time of the initial consultation in the differential diagnosis of azoospermia.

## 2. Materials and Methods

**Figure 1:**
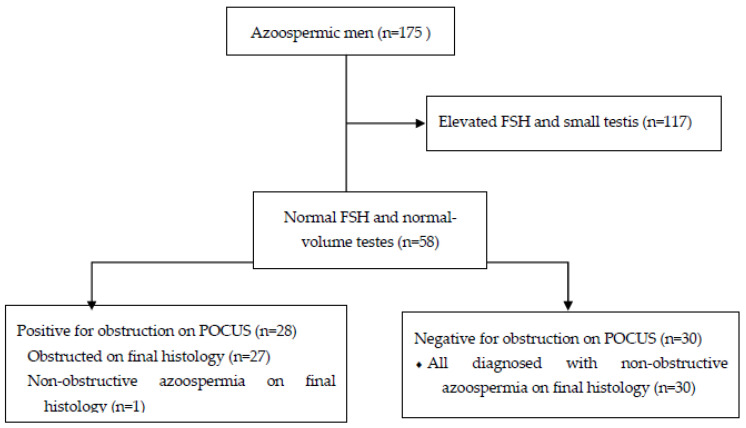
Patients’ characteristics.

The records of 175 consecutive patients who had previously been diagnosed with normal-volume, normal-PH azoospermia and who had undergone surgical sperm retrieval between December 2019 and February 2024 were retrospectively reviewed (Figure 1). A single surgeon (S.B.) performed the surgery in a single fertility center. Azoospermia was confirmed by two semen analyses based on WHO criteria [7] and two extended sperm searches, as described by Ron-El et al. [8]. The diagnosis of NOA was based on the presence of two or more of the following criteria: histopathology that confirmed defective spermatogenesis, the combination of an elevated FSH level > 7.6 IU/L with smaller testicular volume with a long axis of 4.6 cm or less [9], the absence of sperm cells on initial sperm search, and genetic abnormality or chromosomal abnormality known to be associated with NOA (e.g., Klinefelter syndrome; Yq microdeletions). OA was confirmed based on histopathology that confirmed normal spermatogenesis (when available) and the presence of an abundant number of sperm cells on initial sperm search.

**Figure 2:**
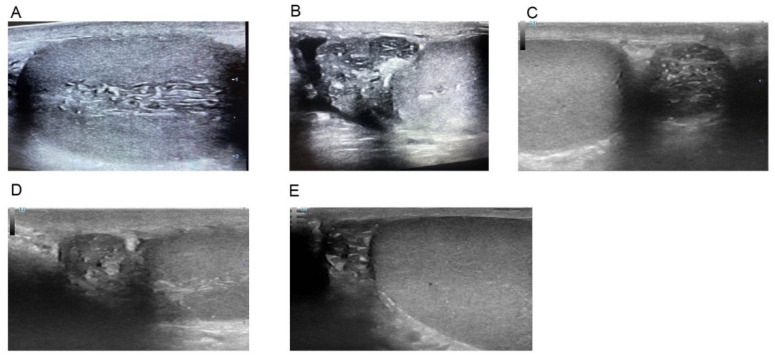
(**A**)—ectasia of the rete testis, (**B**–**E**)—dilation of the epididymal ductules.

**Figure 3:**
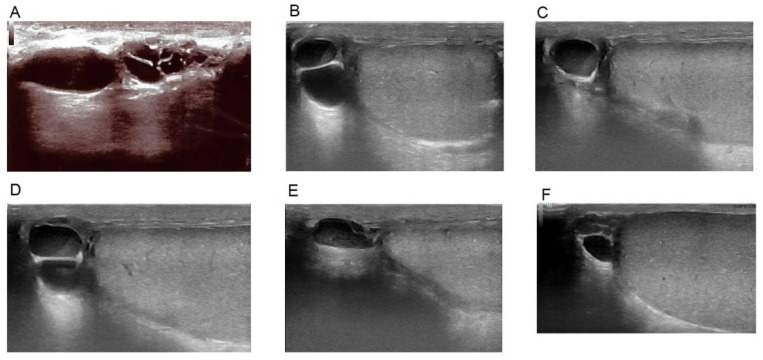
(**A**–**F**) Epididymal cysts with fine internal echoes that were indicative of a spermatocele.

**Figure 4:**
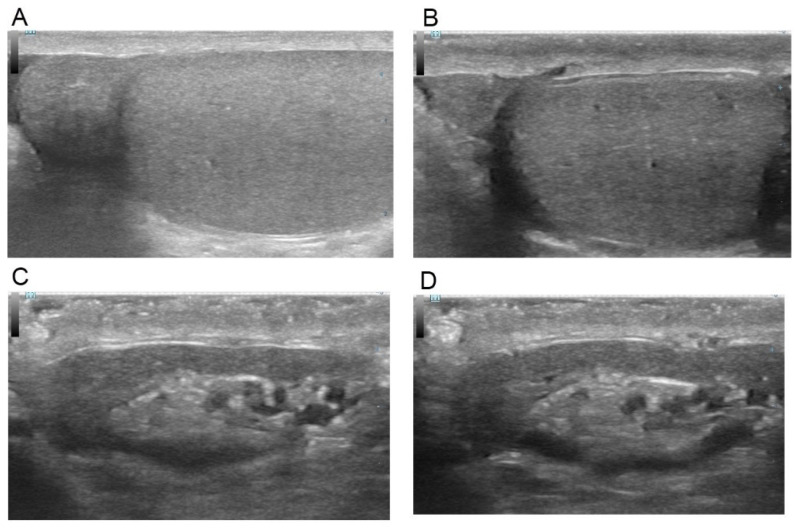
Normal, non-dilated epididymis. (**A**,**B**)—Epididymal head. (**C**,**D**)—Epididymal body and tail.

Patients’ preoperative evaluations included a detailed history and physical examination and a routine scrotal POCUS that was performed during the initial consultation by a single andrologist with 15 years of experience in bedside testicular ultrasound (S.B.) in a clinical setting. POCUS utilized a high-frequency, linear-array transducer with the patient in a supine position. For each patient, both longitudinal and transverse sonograms were obtained for the epididymis and testis. Scrotal images were recorded systematically and categorized as either “secondary signs of obstruction identified” or “secondary signs of obstruction not identified.” The secondary signs of obstruction were defined to include ectasia of the *rete testis* (Figure 2), dilation of the epididymal ductules (Figure 3), which appeared as linear hypoechoic channels, and the presence of epididymal cysts with fine internal echoes that were indicative of a spermatocele (Figure 4).

Hormonal evaluation included serum levels of testosterone, luteinizing hormone (LH), and FSH. All patients underwent karyotyping and Y-chromosome microdeletion analysis.

Blood samples were collected in serum tubes between 7:00 and 10:00 a.m. after 8–10 h of overnight fasting. The same laboratory and kits were used for all samples. FSH, LH, and testosterone levels were analyzed and quantified using a Siemens Advia Centaur XP.

Based on their preoperative assessments, patients with suspected OA were guided to undergo either testicular sperm aspiration (TESA)/microsurgical sperm aspiration (MESA) procedures and those with suspected NOA underwent microdissection testicular sperm extraction (micro-TESE). A total of 28 patients underwent TESA/MESA according to the patient’s preference, and 147 patients underwent micro-TESE. All these patients were included in this study.

### 2.1. Micro-TESE Procedure

Micro-TESE procedures were all performed by a single surgeon (S.B.). Following patient preparation and the induction of general anesthesia, a skin cut was performed on the scrotal median raphe. The testicular envelopes of the larger testicle were opened, and a single testis was delivered through the incision. The tunica albuginea was incised widely in the equatorial plane, and microdissection was performed under a ×20 magnification surgical operating microscope, as originally described by Schlegel [10].

A systematic examination of testicular seminiferous tubules was then performed looking for dilated, opaque tubules. Once identified, tubules were removed using tissue micro-forceps and transferred to the embryology lab for further mechanical disintegration, followed by an initial sperm search. In cases where no thick or opaque tubules were found, systematic multiple micro-biopsies were taken from the open testicle, which encompassed both upper and lower cut surfaces of superficial and deep areas.

Adequate hemostasis was achieved throughout the whole procedure using low-energy bipolar electrocautery. Once the testis was dissected sufficiently, the tunica albuginea was closed using running Vicryl 5/0 sutures. The tunica vaginalis was then closed with running Vicryl 4/0 sutures, and the testicle was placed back into its hemi-scrotum. If no sperm was found on the initial search, a similar procedure was performed on the contralateral testicle.

The dartos layer was closed with running Vicryl 4/0 sutures, and skin edges were approximated with interrupted Vicryl Rapid 4/0. Cord block was performed using 20 mL 0.25% Bupivacaine solution.

### 2.2. Percutaneous TESA Procedure

Following patient preparation, local cord block, and stabilization of the testicle, a needle was inserted along the testicular long axis. The needle was then redirected repeatedly with gentle pressure and aspiration until tubules were sufficiently disrupted and an adequate number of tubules was obtained.

### 2.3. MESA Procedure

Following patient preparation and the induction of general anesthesia, we made a skin cut on the scrotal median raphe. Testicular envelopes of the larger testicle were opened, and a single testis was delivered through the incision. Using an operating microscope, individual epididymal tubules were identified and then aspirated sequentially until an optimal quantity and quality of sperm were obtained. Puncture sites were then closed or cauterized.

### 2.4. Ethics Statement

The present study’s protocol was reviewed and approved by our Institutional Review Board (approval No. 2023001).

## 3. Results

This study included 175 azoospermic men with normal semen volume and pH levels and who had both vas deferens intact upon physical examination. On physical examination, only one man had indurated epididymis suspicious for obstruction. During initial consultation, all men underwent scrotal POCUS examinations. All 147 men who were eventually diagnosed with NOA had no secondary signs of obstruction based on their scrotal POCUS. Sperm was retrieved successfully in 97 men (66%), and retrieval failed in 50 (34%) (Table 1). We then identified the cohort of men with normal FSH levels and normal testicular volume.

We identified 58 patients with normal FSH levels (≤12 IU/L as previously reported, see Table 2) [11]. Thirty of them had no secondary signs of obstruction on scrotal POCUS and subsequently underwent micro-TESE. All 30 men were confirmed to have NOA. On histopathology, 18 (60%) of them were diagnosed with maturation arrest and 12 (40%) with sertoli cell only. Twenty-eight patients demonstrated at least two secondary signs of obstruction, which included ectasia of the rete testis, dilation of the epididymal ductules, and the presence of multiple epididymal cysts observed during scrotal POCUS. Of them, 15 underwent TESA, and 13 underwent MESA procedures. Twenty-seven patients were confirmed to have OA, and one was confirmed as having NOA. On histopathology, he was diagnosed with maturation arrest. Among this cohort of men, the sensitivity of scrotal POCUS in diagnosing OA was 100%, whereas the specificity was 96.8%. Positive predictive value (PPV) and negative predictive value (NPV) were 96.4 and 100%, respectively.

When using even stricter criteria of FSH < 7.6 IU/L, we identified 38 patients (Table 3). Fourteen of them had negative POCUS and underwent micro-TESE, and all were confirmed to have NOA. The other 24 patients had positive POCUS and underwent TESA or MESA, and all were confirmed to have OA.

## 4. Discussion

Our results showed that scrotal POCUS was highly reliable in differentiating OA from NOA in normal-volume, normal-PH azoospermic patients, with a PPV of 96.4% and an NPV of 100%.

Identifying whether azoospermia is obstructive or non-obstructive early in the diagnostic process may enable clinicians to provide a more precise guidance to patients and to direct them towards the appropriate treatment path. Patients with suspected obstructive azoospermia may be guided to undergo a reconstructive procedure or to proceed with surgical sperm retrieval, such as TESA or MESA, and subsequently intracytoplasmic sperm injection (ICSI). For those who are likely to be non-obstructive, counseling about their prospects for successful surgical sperm retrieval should precede their management, which would typically involve micro-TESE followed by in vitro fertilization (ICSI). The possibilities of using donor sperm or adoption are generally introduced as alternatives in case no sperm is recovered. Very early differentiation between obstructive and non-obstructive azoospermia, ideally on initial consultation, can help clinicians reduce the anxiety of those suspected of having obstructive azoospermia by discussing the high likelihood of successful sperm retrieval and more accurately guiding them towards their anticipated diagnosis.

Although diagnosis is easy when FSH is elevated and testicular volume is low, it becomes more difficult with normal levels of FSH and normal testicular volume. AUA/ASRM guidelines even consider a testis biopsy in men with normal FSH and testicular volume to determine the etiology [4].

Ultrasonography (US) is considered the gold-standard imaging approach for scrotal investigation [12]. It can identify changes in the size and echotexture of the testes and provide information on epididymis-related abnormalities [13].

A systematic review on the diagnostic accuracy of scrotal US in the evaluation of infertile men clarified that epididymis head and/or tail dilation was suggestive of obstruction or inflammation of the male genital tract. In addition to abnormalities in the echo pattern, obstruction or inflammation were related to impaired sperm parameters [14].

With US, the normal epididymal head is triangular, with echogenicity comparable with that of the testis and usually slightly more echogenic than the body and tail [15,16]. A dilated epididymis associated with echo-pattern abnormalities, which include calcifications, may represent past infection/inflammation [14,15,16]. Dilation of the epididymal ductules, which appear as linear hypoechoic channels, and the presence of multiple epididymal cysts with fine internal echoes may suggest post-testicular obstruction [17,18].

POCUS is a widely used bedside tool that aids in clinical diagnoses [6]. It enables the treating clinician to immediately integrate sonographic imaging into the clinical evaluation, correlating the findings with the patient’s presentation. Affordable handheld ultrasound systems are now widely available, and there is evidence that non-radiologists can become competent in the performance of POCUS [19,20]. POCUS is now used in many practice settings, including screening, diagnosis, procedural guidance, and monitoring, and it has become associated with changes in clinical decision-making in medical practice [21].

The interest in a noninvasive approach to distinguish between OA and NOA at an early stage has prompted various studies. These studies have shown that MRI, through the identification of specific parameters, can differentiate between the two diagnoses [22]. However, MRI is considerably more expensive and time-consuming than the simpler method we describe here. Furthermore, early identification of OA or NOA by an andrologist can direct attention to specific findings in advanced genetic analyses, like whole-exome sequencing, potentially enhancing diagnostic precision and treatment outcomes [23].

With the introduction of sonographic contrast agents, contrast-enhanced ultrasound (CEUS) has been studied for morphological characterization and local staging of different testicular diseases. In the last few years, studies have described the advantages of CEUS, especially its role in the noninvasive identification of testicular masses [24]. In the near future, we may find a role for CEUS in the evaluation of infertile men. However, at this point, data are scarce [25].

We found that POCUS was highly effective and reliable in identifying obstructive azoospermia at the time of the initial consultation when used by a non-radiologist treating clinician. To the best of our knowledge, this study is the first to introduce the use of scrotal POCUS in andrology, particularly to investigate its reliability for the differential diagnosis of azoospermia.

We recommend integrating scrotal POCUS into the initial evaluation of patients with azoospermia. This strategy could facilitate a faster and more precise preoperative distinction between OA and NOA. The advantages of this approach include diminishing patient stress and enabling a more targeted treatment strategy. Because the effectiveness of ultrasound is operator-dependent, it is essential that clinicians (andrologists/urologists) receive proper training and use ultrasound regularly in clinical settings. This approach is crucial for enhancing the diagnostic accuracy and reliability of scrotal POCUS.

For scrotal POCUS training programs, we believe that such training should include a minimum of basic knowledge in ultrasound physics, supervised sessions of image acquisition, and their interpretation.

Our main limitation was the retrospective nature of this study, which may expose the results to several biases. However, the fact that POCUS was performed consecutively on every patient lowers the chances of major biases.

## 5. Conclusions

Scrotal POCUS is an effective clinical diagnostic tool for distinguishing obstructive and non-obstructive azoospermia. Because it is noninvasive, safe, and affordable, it is an ideal bedside clinical tool that can serve the skilled non-radiologist clinician reliably.

## Figures and Tables

**Table 1:** Clinical characteristics of 147 men who were diagnosed with NOA.

		N	%	Retrieved		AGE		FSH		LH		Testosterone	
				N	%	Mean	SD	Mean	SD	Mean	SD	Mean	SD
Diagnosis	UNEXPLAINED (POCUS)	74	50	41	55.4	32.2	5.9	21.8	13.3	9.0	5.3	15	7.2
	Klinefelter’s	39	26.5	30	76.9	27.5	5.7	39.2	13.8	21.4	7.5	7.9	3.7
	Undescended testis	11	7.5	8	72.7	31.5	6.3	24.7	13.7	11.4	6.0	13.9	7.4
	Y microdeletion	11	7.5	8	72.7	32.0	6.8	16.4	8.7	7.0	2.4	12.6	5.5
	Post chemo	9	6.1	7	77.7	32.2	6.3	25.0	10.4	12.8	6.9	13.2	4.8
	Translocation	2	1.4	2	100	24.5	0.7	8.1	5.4	4.1	2.4	16.8	3.5
	45XO mosaic	1	0.7	1	100	39	N/A	13.5	N/A	5	N/A	13.2	N/A

**Table 2:** Azoospermic men with normal semen volume and pH levels. FSH ≤ 12.

	N	%	Retrieved		AGE		FSH		LH		Testosterone	
			N	%	Mean	SD	Mean	SD	Mean	SD	Mean	SD
POCUS+	28	48.2	28	100	31.6	7.8	5.8	2.6	4.4	1.2	15.1	5.0
POCUS−	30	31.0	19	63.3	31.1	6.5	7.3	5.7	5.1	1.9	14.2	8.1

**Table 3:** Azoospermic men with normal semen volume and pH levels. FSH < 7.6.

		N	%	Retrieved		AGE		FSH		LH		Testosterone	
				N	%	Mean	SD	Mean	SD	Mean	SD	Mean	SD
Diagnosis	UNEXPLAINED (POCUS+)	24	63.1	24	100	31.5	7.3	5.0	1.5	4.2	1.2	14.5	4.2
	UNEXPLAINED (POCUS-)	10	26.3	6	60	29.8	8.0	5.9	2	3.4	1.8	16.7	10.1
	Undescended testis	1	2.6	1	100	30	N/A	7	N/A	5	N/A	8	N/A
	Y microdeletion	2	5.3	1	50	31	1.4	5.7	0.8	4.4	0.7	12.1	2
	Translocation	1	2.6	1	100	25	N/A	4.3	N/A	2.4	N/A	19.3	N/A

## Data Availability

All Data will be made available on request from the corresponding author.
